# British Medical Temperance Association

**Published:** 1887-10

**Authors:** 


### The British Medical Temperance Association.

One of the curious features of the British Medical Association may be observed at the annual dinners. The price of membership in this body is one guinea. Those who desire to attend the annual dinner may do so for an additional guinea. In connection with the annual meetings, there is what has been styled the British Medical Temperance Association. Those who take no wine at the annual dinner may have tickets at seventeen shillings each. These tickets are printed on blue card, and as the ticket must lie in front of each plate, the servants readily see who have the blue ticket, and are, therefore, enabled to avoid giving offense to members of the temperance association by offering them wine.

Total abstinence is what the blue card means, yet temperance is what the possessor usually claims. The attempt to make this distinction and to have it better understood has led to some controversial correspondence, in which Mr. James Ridge, the honorable Secretary of the B. M. T. A., announces that temperance is far wider in its meaning than total abstinence. He insists the object of the organization is to confirm and increase self-control, by the agency of total abstinence from certain liquors containing alcohol, whose physiological action is to diminish self-control and finally to abolish it. Mr. Ridge thinks alcohol in any form the material cause of moral evil, and that the doctor of the body should be the first to raise his voice, and thus co-operate with the doctors of the soul to elivate the race and raise the moral standard of the nation.

